# Evolutionary Transition in the Late Neogene Planktonic Foraminiferal Genus *Truncorotalia*

**DOI:** 10.1016/j.isci.2018.09.013

**Published:** 2018-10-17

**Authors:** Russell D.C. Bicknell, Katie S. Collins, Martin Crundwell, Michael Hannah, James S. Crampton, Nicolás E. Campione

**Affiliations:** 1 Palaeoscience Research Centre, School of Environmental and Rural Science, University of New England, Armidale 2351, Australia; 2 Department of the Geophysical Sciences, University of Chicago, 5734 South Ellis Avenue, Chicago, IL 60637, USA; 3 Department of Paleontology, GNS Science, Lower Hutt 5040, New Zealand; 4 School of Geography, Environment and Earth Science, Victoria University of Wellington, Wellington 6140, New Zealand

**Keywords:** Ecology, Biological Sciences, Evolutionary Biology, Phylogenetics, Paleobiology

## Abstract

The fossil record provides empirical patterns of morphological change through time and is central to the study of the tempo and mode of evolution. Here we apply likelihood-based time-series analyses to the near-continuous fossil record of Neogene planktonic foraminifera and reveal a morphological shift along the *Truncorotalia* lineage. Based on a geometric morphometric dataset of 1,459 specimens, spanning 5.9–4.5 Ma, we recover a shift in the mode of evolution from a disparate latest Miocene morphospace to a highly constrained early Pliocene morphospace. Our recovered dynamics are consistent with those stipulated by Simpson's quantum evolution and Eldredge-Gould's punctuated equilibria and supports previous suppositions that even within a single lineage, evolutionary dynamics require a multi-parameter model framework to describe. We show that foraminiferal lineages are not necessarily gradual and can experience significant and rapid transitions along their evolutionary trajectories and reaffirm the utility of multivariate datasets for their future research.

## Highlights

• Evolution of planktonic foraminiferal anatomy across Miocene/Pliocene boundary
• Novel application of multivariate analyses to 1,459 specimens
• Evidence for a punctuated anatomical shift associated with the boundary

## Graphical Abstract

**Figure:**
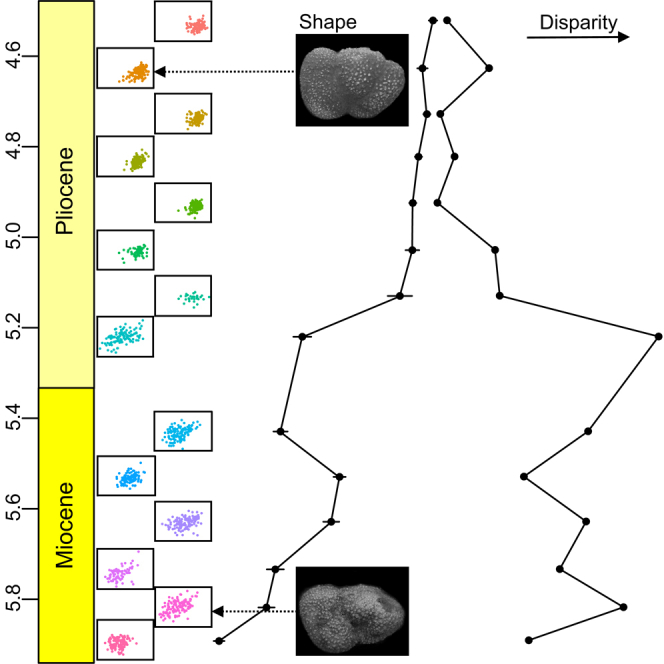

## Introduction

Reconstruction of evolutionary patterns, such as gradualism, stasis, quantum evolution (QE), punctuated equilibria (PE), and punctuated anagenesis, have driven generations of evolutionary biologists and palaeontologists to understand the history of life (e.g., Darwin, 1859, Depéret, 1907, Simpson, 1944, Eldredge and Gould, 1972, Gould and Eldredge, 1977, Wei and Kennett, 1988, Hunt, 2006, Hunt et al., 2015). Many fossil groups have been used to document evolutionary trends (see Hunt, 2007). Among them, the planktonic foraminiferal fossil records are recognized as being especially useful for studying the tempo and mode of evolution (Frerichs, 1971, Malmgren et al., 1983, Stanley et al., 1988, Collins, 1989, Hunt, 2006, Ezard et al., 2011, Pearson and Ezard, 2014). Planktonic foraminifera are single-celled marine protists with calcite shells (or tests) that are distributed almost ubiquitously in the ocean (Strotz and Allen, 2013, Hsiang et al., 2016). Tests are structurally robust and easily preserved in hemipelagic sediments and biogenic oozes in the deep sea, which are not typically affected by high rates of erosion (Aze et al., 2011). Under ideal sedimentation conditions, these deposits represent specimen-rich, near-continuous records of deposition (Pearson, 1993, Aze et al., 2011, Lazarus, 2011). The ability to gather large samples of specimens at a high temporal resolution makes deep-sea microfossil records ideal for studying and understanding evolution (Lazarus, 2011).

Neogene planktonic foraminiferal fossil lineages have been used to interpret gradualism (Arnold, 1983, Belyea and Thunell, 1984, Wei, 1987, Wei and Kennett, 1988), PE (Wei and Kennett, 1988), and punctuated anagenesis (Malmgren et al., 1983, Malmgren et al., 1996). However, the last decade has seen the emergence of sophisticated model-fitting techniques for time series that are ideal tools for testing the evolutionary tempo and mode (Hunt, 2006, Hunt, 2008, Hunt and Carrano, 2010, Hunt et al., 2015). These advances call for a re-evaluation of the previously interpreted evolutionary patterns, and a consideration of understudied late Neogene lineages, such as *Truncorotalia*, examined here. Recent truncorotalid diversity is related to the evolution of *Truncorotalia crassaformis*, an extant species that arose after the Miocene/Pliocene boundary from a contentious ancestral species (Hornibrook, 1981, Kennett and Srinivasan, 1983, Cifelli and Scott, 1986, Bylinskaya, 2004, Boudagher-Fadel, 2012, Scott et al., 2015). Notably, Arnold (1983) hypothesized a gradual transition from *Truncorotalia juanai* (=*Hirsutella cibaoensis* in Arnold (1983)) toward *T. crassaformis* across the boundary. However, by using semilandmark geometric morphometrics and maximum likelihood-based time-series analyses (Hunt et al., 2015) we reveal an abrupt evolutionary transition along the *Truncorotalia* lineage after the Miocene/Pliocene boundary (Crundwell and Nelson, 2007). Our results therefore contradict previous theories and preclude the need for an intermediate form along the transition (*sensu* Cifelli and Scott, 1986).

## Results

**Figure 1:**
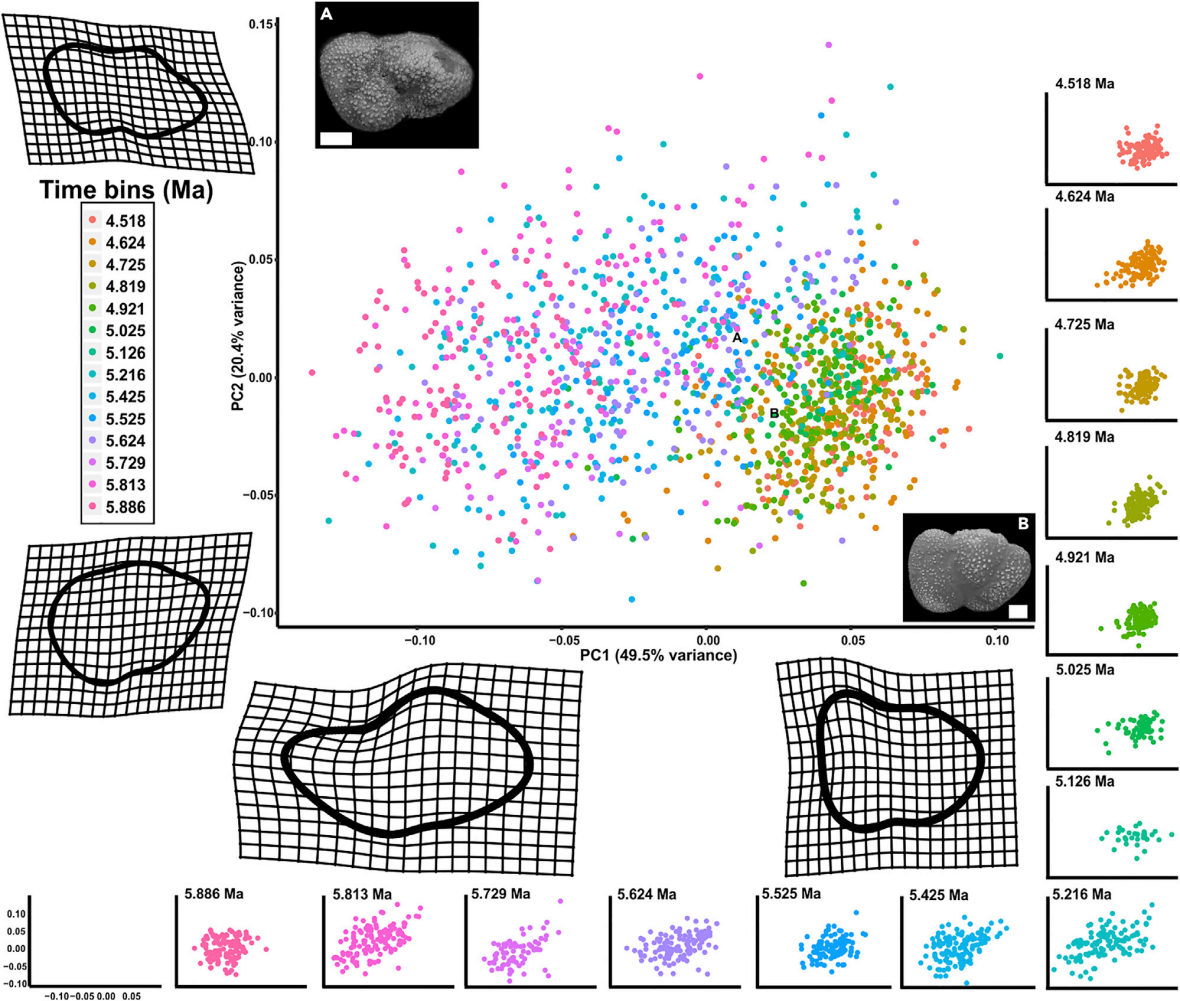
Evolution of *Truncorotalia* in Principal Component Analysis Space Clusters migrate from negative to positive PC1 values across the studied intervals. This shift reflects a taxonomic change associated with the inflation of the final chamber and is typified by a large jump in morphology at about 5.1 Ma. Note that there is no time bin for 5.3 Ma due to the lack of specimens. (A) *Truncorotalia juanai* specimen (Specimen FP5584). (B) *Truncorotalia crassaformis* specimen (Specimen FP5583). Scale bars: 50 μm in (A and B). Scanning electron micrographs were taken using a JEOL JSM-6610LA at Victoria University of Wellington, under 15-kV and low vacuum. Specimens were not coated before imaging. Specimens are housed at GNS Science, Lower Hutt 5040, New Zealand.

**Figure 2:**
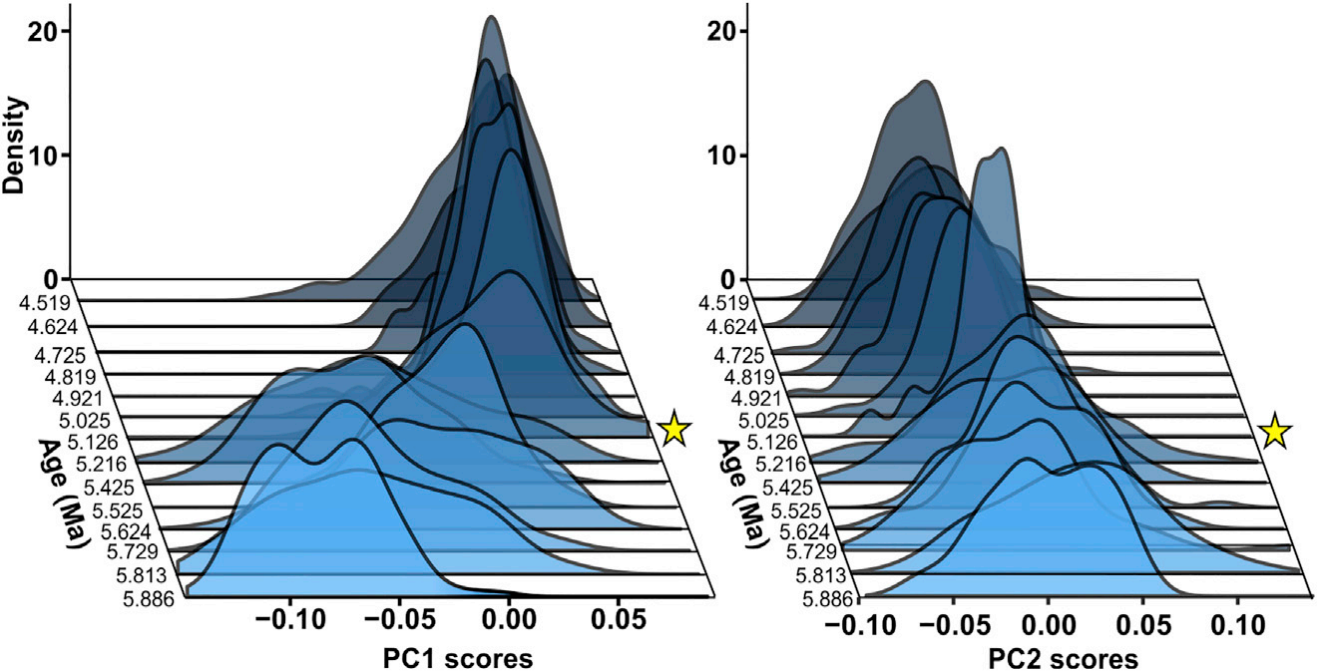
Transitions of Truncorotalid Morphospace Defined by PC1 and PC2 The transition from a diverse population of *Truncorotalia* to a constrained *Truncorotalia* population is most obvious at 5.1 Ma in PC1 and is present, but more subtle, in PC2. Stars indicate where the transition occurs.

**Figure 3:**
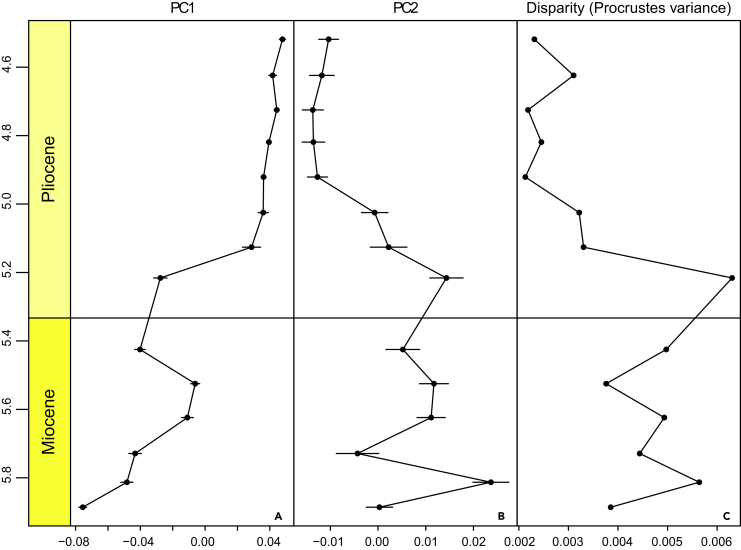
Mean Morphology and Disparity of Truncorotalids across the Miocene/Pliocene (A) PC1 shows a jump from an unbiased random walk to stasis at 5.1–5.2 Ma. Error bars reflect the variance in each time-bin. (B) PC2 shows a contemporaneous but less pronounced shift than PC1. PC2 is best described by a more punctuated model. Error bars reflect the variance in each time-bin. (C) Disparity through time reveals an associated shift in the range of morphological variation at 5.1–5.2 Ma, demonstrating the rapid fixation of the novel morphology following the shift. Related to Tables S1–S3.

Principal component (PC) 1, derived from a Procrustes-based geometric morphometric analysis, describes the degree of test ventral inflation (49.5% of total variance). *Truncorotalia crassaformis*, typically known from Pliocene deposits, has a more ventrally elevated final test chamber compared with the typically Miocene *T. juanai* (Cifelli and Scott, 1986). PC1 therefore defines the major differences between end members of the lineage across this temporal interval and is the most useful for understanding the evolutionary transition within *Truncorotalia* (Figure 1, outline reconstructions). The clusters in each time bin—specimens from sampled horizons in PC space—transition from negative PC1 space to positive PC1 space across the studied interval. There is a jump at 5.1 Ma (Figure 1, time bin plots and Figure 2), which was equally documented in the time-series plots (Figures 3A and 3B). Furthermore, this shift in morphology is associated with a significant ∼50% decrease in the range of morphological variation (disparity) calculated as Procrustes variances (PV)—PV_5.216_ = 0.0063 to PV_5.126_ = 0.0033 (PV distance = 0.003, p value = 0.001; Figure 3C). Overall, between 4.5 and 5.1 Ma disparity values are lower (PV ranges between 0.0021 and 0.0033) and points cluster closer in the PC space than between 5.1 and 5.9 Ma (PV ranges between 0.0038 and 0.0063). Timing of the transition to samples with constrained morphological variation reaffirms the suggested first-appearance datum of 5.1 Ma at Deep Sea Drilling Project (DSDP) Site 593 in Crundwell and Nelson (2007). PC2 tracks the change in test shape from more axially compressed to more axially expanded (20.4% of total variance), similar to the proposed changes in *Truncorotalia* noted in Arnold (1983) and Cifelli and Scott (1986).

**Figure 4:**
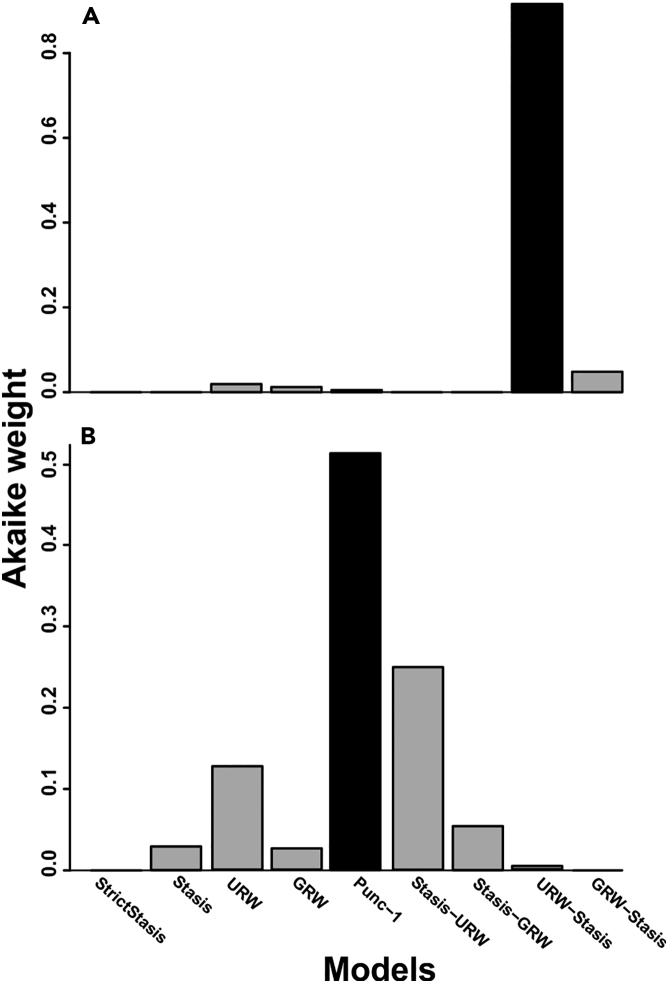
Bar Plots of the Akaike Weights of the Nine Models (A) PC1 is best explained by a shift model of unbiased random walk to stasis. (B) PC2 is best explained by punctuated equilibria model, although other models received some support. Black bars represent the best supported models.

The time-series analyses provide overwhelming evidence of a shift along PC1 (Figure 4A; Table S2). A comparison of nine models supports only an unbiased random walk to stasis shift model (Akaike weight [AW] = 0.916). All other models are rejected, including the generalized random walk model (AW = 0.012) needed to support a hypothesis of gradual phyletic change over time. Support values for PC2 also suggest a shift (Figure 4B), either between static intervals (i.e., PE model; AW = 0.512) or from an unbiased random walk to stasis (AW = 0.249). However, a simple unbiased random walk across the whole time series along PC2 also received some support compared with other models (AW = 0.127) (Table S3). No statistical support for bimodality in any time bin along PC1 and PC2 was found (Data S5).

## Discussion

The origin of recent truncorotalid diversity is traced back to the evolutionary event documented across the Miocene/Pliocene boundary considered here (Kennett and Srinivasan, 1983, de Vargas et al., 1999, Boudagher-Fadel, 2012, Scott et al., 2015). *Truncorotalia crassaformis* evolved abruptly after the Miocene/Pliocene boundary from a contentious ancestral species (Hornibrook, 1981, Cifelli and Scott, 1986, Bylinskaya, 2004, Scott et al., 2015). Uncertainty regarding the ancestral taxon has arisen as the appearance of *T. crassaformis* has not been substantiated with a “well-authenticated evolutionary sequence” (Cifelli and Scott, 1986, p. 50). *T. juanai* (Scott et al., 1990, Crundwell and Nelson, 2007), *Hirsutella cibaoensis* (Arnold, 1983, Aze et al., 2011, Boudagher-Fadel, 2012)*, T. crassula* (Kennett and Srinivasan, 1983)*, Globorotalia aemiliana* (Colalongo and Sartoni, 1967, Lamb and Beard, 1972), *G. subscitula* (Blow, 1969), and a “nonspecialized scituline globorotalid” (Cifelli and Scott, 1986, p. 49) have all been suggested as possible ancestral taxa. Here we developed on Cifelli and Scott (1986) and others to support the thesis that *T. juanai* (=*H. cibaoensis* in Arnold (1983)) experienced a rapid evolutionary transition to the ventrally inflated *T. crassaformis*. This change lacked a transitional *T. crassaformis* population (i.e., metaspecies) along the temporal sequence, although it is characterized by a peak in disparity immediately before the transition where both *T. juanai* and *T. crassaformis* morphologies were present (Figures 1 and 4). Here we interpret the patterns within the context of two phyletic evolutionary theories, PE and QE, but acknowledge that extinction-survival dynamics cannot be precluded. This interpretation rejects the notion of a gradual transition along the *Truncorotalia* lineage proposed by Arnold (1983). Although Arnold (1983) documented an accelerated rate of evolution along the lineage in the early Pliocene, his broader interpretation was likely biased by the prevailing opinion at that time: that planktonic foraminiferal evolution was predominantly gradual (Wei, 1987, Wei and Kennett, 1988, Aze et al., 2011).

### Punctuated Equilibria

The theory of PE was presented by Eldredge and Gould (1972) as an alternative to phyletic gradualism and drove extensive research evaluating various evolutionary patterns and processes in the fossil record in search of evolutionary stasis (e.g., Stanley, 1979, Gingerich, 1985, Turner and Paterson, 1991, Jackson and Cheetham, 1999, Hunt et al., 2015). PE predicts that evolutionary dynamics can be described by long periods of stability that are punctuated by rapid pulses of change. Morphological variation accumulated within a lineage is therefore normally low, and the majority of variation is produced during rapid, episodic events (Eldredge and Gould, 1972, Eldredge et al., 2005). These events occur so rapidly that they are seldom recorded in the fossil record (Eldredge and Gould, 1972, Eldredge et al., 2005). The fundamental tenet of PE is therefore stasis. This feature of PE is difficult to define, and stasis is therefore generally referred to as “little to no net accrued speices-wide morphological change” (Eldredge et al., 2005, p. 133). Mathematical definitions of stasis include (1) random variance over time, which assumes that the morphological conditions at one time interval are independent of those of the preceding or succeeding interval (“stasis” model of Hunt et al. (2015)), and (2) highly constrained variance that approaches zero (“strict stasis” model of Hunt et al., (2015)). Even unbiased random walks—variation in morphological change between intervals about a steady mean—can also describe little to no net change (Eldredge et al., 2005).

With these stipulations in mind, we posit that our results may conform to the predictions made by PE. This is especially the case for PC2, which recovers equivocal support for the “Punc-1” model of Hunt et al. (2015)—stasis before and after a shift. PC1 shows support for stasis after the transition and an unbiased random walk before the transition. This pattern does not conform to the traditional view of PE (Eldredge and Gould, 1972, Gould and Eldredge, 1977, Kirkpatrick, 1982); however, stasis as defined by Eldredge et al. (2005) could include the basic patterns characteristic of an unbiased random walk. *Truncorotalia* may have therefore experienced PE after the Miocene/Pliocene boundary.

### Quantum Evolution

Three decades before Eldredge and Gould's contribution, George G. Simpson proposed QE: a rapid shift from one adaptive zone to another (Simpson, 1944, Simpson, 1953). Simpson considered QE as one end member of a spectrum within phyletic evolution (i.e., ancestor-descendent relationships; Simpson, 1953) with phyletic gradualism at the other end. Simpson (1953) particularly viewed QE as the process by which higher taxonomic levels (such as families and orders) originated and associated the theory with the initial explosive phase of adaptive radiations. Although QE has been considered outdated, especially regarding the “evolution” of higher taxonomic ranks, it makes certain predictions about the nature of an evolutionary (quantum) shift that PE does not. QE therefore remains a viable explanation for evolutionary patterns and was even recently interpreted as a mechanism in the adaptive radiation of bird bills (Cooney et al., 2017). As such, we have highlighted this hypothesis as an alternative to PE.

The observed pattern of evolution from morphologies characteristic of *Truncorotalia juanai* to those of *T. crassaformis* could be explained as a quantum evolutionary shift along the planktonic foraminiferal lineage given that we recover a rapid (tachytelic), “linear, but relatively short” (Simpson, 1944 p. 216), and “all-or-none” (Simpson, 1953 p. 389) transition from an ancestral morphology to a descendant morphology. The ancestral group—*T. juanai—*does not persist after *T. crassaformis* evolved (Simpson, 1944, Simpson, 1953, Kirkpatrick, 1982). This “sharp shift from one position [morphology] to another” (Simpson, 1944, p. 216) is documented in Figure 2 and partly mirrors the idealized representation of QE presented in Simpson (1944, Figure 31) and Kirkpatrick (1982, Figure 3) and can certainly be interpreted within the phyletic evolution continuum of Simpson, 1953. Although aspects of this shift also conform to the stipulations of PE (see previous section), our results reveal that *T. juanai* was a morphologically diverse species during the Miocene before an evolutionary event in the Pliocene, where the descendant species became morphologically constrained (Kirkpatrick, 1982, Arnold, 1983, Cifelli and Scott, 1986) (Figure 3). Associated transitions between morphology and disparity are not explicitly required for PE, but are a stipulation of QE. Specifically, we recover “… relative instability with the system shifting toward an equilibrium not yet reached” and fluctuations in variation and a “new variant [that is] … rapidly fixed” (Simpson, 1944; table 19). These dynamics support the idea that QE might explain the major and abrupt evolutionary shift within *Truncorotalia* after the Miocene/Pliocene boundary, perhaps in preference to PE.

One important expectation of QE remains difficult to infer: the shift after the Miocene/Pliocene boundary represents a transition from one adaptive zone to another. As described earlier, the morphological shift is associated with a ventral extension of the final test (Figures 1, 2, and 3). In Neogene planktonic foraminifera, ventrally extended test shapes are considered to be adaptations to better exploit oligotrophic waters, based on the observed distribution of ventrally inflated extant groups (Scott et al., 1990, Scott et al., 2015). Furthermore, extant *Truncorotalia* species are deep-water dwelling, ventrally inflated species that inhabit a deep thermocline (Hornibrook, 1981, Boudagher-Fadel, 2012). Selection for a subset of the *Truncorotalia* population about the Miocene/Pliocene boundary may therefore have produced a morphological stock capable of adapting to a deep-oceanic zone, eventually resulting in the recent truncorotalid diversity (de Vargas et al., 2001, Aze et al., 2011, Quillevere et al., 2011, Scott et al., 2015). Indeed, numerous studies have linked changes in planktonic foraminiferal morphology to shifts in adaptive zones and responses to environmental conditions (e.g., de Vargas et al., 1999, Quillevere et al., 2011). However, without comparisons to other lineages and a more detailed assessment of the paleoenvironments from DSDP Site 593, the driver(s) behind this transition and associated adaptive responses, if present, remains speculative.

### Future Directions

The first recorded appearance of *Truncorotalia crassaformis* in the fossil record is at 5.7 Ma (Aze et al., 2011), before the shift recognized here. Such a scenario would predict a bimodal distribution in older samples illustrating the co-existence of both species (*sensu* Scott, 2011), rejecting phyletic evolution, and supporting the extinction of *T. juanai* and survival of *T. crassaformis*. However, statistical tests for bimodality failed to reject unimodality in any time bin along PC1 and PC2 (Data S5). Furthermore, morphologies typical of *T. crassaformis* (positive PC1 and negative PC2 values; lower left quadrant) are exceedingly rare in horizons before the shift. Such morphologies account for a maximum of 13% of the population in any given Miocene sample, but account for ∼27%–68% of populations in Pliocene samples (Table S4). These data both suggest that *Truncorotalia* populations during the Miocene exhibit high levels of disparity, sometimes even expanding the morphological gamut into regions similar to *T. crassaformis*. However, true *T. crassaformis* appear abruptly and are quickly fixed to a largely confined area of morphospace. Future research could expand the temporal scope of our analyses, older than 5.9 Ma, which may provide greater context for the onset of morphologies associated with *T. crassaformis*. Furthermore, a study using another DSDP site preserving material that spans 5.8–4.5 Ma and that is geographically separated from DSDP Site 593 could determine if the evolutionary event recovered here is general or localized (*sensu* Lazarus et al., 1995).

### Limitations of Study

Two uncertainties concerning the materials used here are noted. First, the evolutionary transition occurred at the change from DSDP Site Hole 593 to Hole 593A residues. Although unfortunate, this would not have impact on the recovered evolutionary patterns, as both holes were obtained from the same site (DSDP 593); were drilled less than 10 m apart, from the same longitude and latitude; and have been treated as duplications of each other by previous workers (Kennett and von der Borch, 1986). Despite their proximity, we cannot completely reject the possibility of a migratory event between the two holes. However, we view this option as unlikely as planktonic foraminifera are generally well distributed within major bodies of water (Cifelli and Scott, 1986) and no major changes in oceanic waters masses occurred at DSDP Site 593 across the Miocene/Pliocene boundary (Nelson and Cooke, 2001). A further control on planktonic foraminiferal distribution is vertical niche portioning within the water column (Seears et al., 2012). This portioning would have allowed populations to be separated, but would not have prevented the record of *Truncorotalia crassaformis* at DSDP 593. As such, although a migration event is possible, it seems unlikely. As mentioned above, a study of another hole would help explore this issue further.

The second uncertainty is the notable loss of specimens between 5.2 and 5.3 Ma, before the shift. No typical *Truncorotalia* specimens are recovered from this interval, although rare, malformed individuals (i.e., specimens with aberrant morphologies) were found (Mancin and Darling, 2015). Although malformed individuals were not included in this study, as we wanted to assess the transition within a standard population, it is worth noting that malforms reflect stressed populations and an abiotic response to oceanic conditions (Mancin and Darling, 2015, Kontakiotis et al., 2016). Oceanic cooling at the terminal Miocene may have affected the *Truncorotalia* population, producing malforms, hinting at potential drivers behind the evolutionary events described here (Hornibrook, 1981, Malmgren and Berggren, 1987, Stanley et al., 1988, Lear et al., 2015).

### Conclusion

We documented and assessed the evolutionary transition along *Truncorotalia* across the Miocene/Pliocene boundary using semilandmark morphometrics and time-series analyses. A potentially localized and rapid evolutionary shift between two end members of *Truncorotalia*, *T. juanai* and *T. crassaformis*, at 5.1–5.2 Ma reveals that the evolutionary dynamics were not gradual and rejects the notion of an intermediate form along the lineage (*contra* Arnold, 1983, Cifelli and Scott, 1986). The transition between end members involved a major reduction in morphological diversity and a transition to a more constrained morphological stock. Furthermore, likelihood-based time-series analyses strengthen this hypothesis through rejection of simple gradual or random modes of evolution, in favor of shift models, which can be interpreted within the context of both Simpson's QE and Eldredge and Gould's PE. Through this study we hope to augment research into tempo and mode in planktonic foraminifera and highlight certain expectations of Simpson's theory, which are not explicit to PE. We envision that application of these methods by planktonic foraminiferal researchers will garnish further explicit tests of tempo and mode in this iconic fossil group.

## Methods

All methods can be found in the accompanying Transparent Methods supplemental file.

## Supplemental Information

Document S1. Transparent Methods and Tables S1–S4

Data S1. TSP File of Analyzed Specimens, Related to Figures 1 and 2

Data S2. CSV File for Semilandmark Sliding, Related to Figure 1

Data S3. CSV File for Assigning Age Bins to Specimens, Related to Figure 1

Data S4. Results of Disparity Analysis, Related to Figure 3

Data S5. Results of Hartigan's Dip Test, Related to Figure 2

Data S6. R Code Employed for Analyses, Related to Figures 1, 3, and 4 Notes are included in code to allow for ease of use.
