# Supplementary material for: Evolutionary Transition in the Late Neogene Planktonic Foraminiferal Genus Truncorotalia
Source: iScience. 2018 Oct 17;8:295–303. doi: 10.1016/j.isci.2018.09.013 (PMC6205115; doi:10.1016/j.isci.2018.09.013)
Supplement: Document S1. Transparent Methods and Tables S1–S4 [file mmc1.pdf]

ISCI, Volume *8*

Supplemental Information

Evolutionary Transition in the Late Neogene Planktonic Foraminiferal Genus *Truncorotalia*

Russell D.C. Bicknell, Katie S. Collins, Martin Crundwell, Michael Hannah, James S. Crampton, and Nicolás E. Campione

Time bins	PC1 mean	PC1 variation	PC2 mean	PC2 variation	Number of specimens
4.5185	0.047824677	0.000434703	-0.010357565	0.000507552	118
4.624	0.041749406	0.000663263	-0.011769851	0.000785937	118
4.725	0.044311474	0.000312265	-0.013678819	0.000582694	120
4.819	0.039455399	0.000309778	-0.013549356	0.00066271	118
4.921	0.036157805	0.000304769	-0.012696294	0.000518549	119
5.025	0.035912368	0.000648122	-0.00070798	0.000506534	66
5.126	0.028714004	0.000937043	0.0022205	0.000442058	30
5.216	-0.027622986	0.001992911	0.014342546	0.001455831	119
5.425	-0.040181654	0.00124135	0.005203453	0.001464512	118
5.525	-0.006104463	0.000746298	0.011721271	0.000886289	97
5.624	-0.010927092	0.001594211	0.01113362	0.001051029	120
5.729	-0.04320383	0.001159867	-0.004300164	0.001502031	76
5.813	-0.048301772	0.001659668	0.023662372	0.001701167	120
5.886	-0.075546774	0.000720687	0.000298733	0.000926812	120

Table S1: Summary of the data entered into paleoTS, related to Figure 3.

Model	Ancestral state	Θ1	Θ2	σ2	M	ω	Shift (time bin)	LogL	AIC	AW
**StrictStasis**		0.019						-1700	3414	0.000
**Stasis**		0.002				0.002		24.9	-44.7	0.000
**URW**	-0.075			0.005				35.6	-66.1	0.019
**GRW**	-0.075			0.004	0.090			36.8	-65.3	0.012
**Punc-1**		-0.036	0.039			0	7	37.9	-63.4	0.005
**Stasis-URW**		-0.029		0.001		0.012	7	31.5	-50.5	0.000
**Stasis-GRW**		-0.029		0.037	0.031	0.001	7	28.9	-40.5	0.000
**URW-Stasis**	**-7.51^{-2}**	**3.98^{-2}**		**5.25^{-3}**		**1.79^{-5}**	**7**	**45.7**	**-73.9**	**0.916**
**GRW-Stasis**	-7.51^{-2}	3.96^{-2}		5.07^{-3}	7.15^{-2}	1.81^{-5}	7	46.0	-68.0	0.048

Table S2: Summary data from the paleoTS model fitting for PC1 to 3 significant figures, related to Figure 4. Bold is the preferred model. Θ1=mean one, Θ2=mean 2, σ^2=step variance, μ= step mean, ω=trait variance. LogL=log likelihood, AIC= Akaike information criterion , AW=waited AIC values.

Model	Ancestral state	Θ	Θ2	σ2	M	ω	Shift (time bin)	LogL	AIC	AW
**StrictStasis**		-0.004						-40.6	83.6	0.000
**Stasis**		-8^{-5}				0		42.6	-80.2	0.029
**URW**	0.001			0.002				44.1	-83.1	0.127
**GRW**	0.002			0.001	-0.009			44.2	-79.9	0.026
**Punc-1**		**8.98^{-3}**	**-8.99^{-3}**			**3.98^{-5}**	**7**	**49.2**	**-85.9**	**0.512**
**Stasis-URW**		1.03^{-2}		6.06^{-4}		8.38^{-5}	7	48.5	-84.5	0.249
**Stasis-GRW**		1.03^{-2}		4.08^{-4}	-2.07^{-02}	9.12^{-5}	7	49.4	-81.4	0.054
**URW-Stasis**	0.001	-0.009		0.003		0	7	46.6	-75.6	0.004
**GRW-Stasis**	0.001	-0.009		0.003	0.021	0	7	46.8	-69.6	0.000

Table S3: Summary data from the paleoTS model fitting for PC2 to 3 significant figures, related to Figure 4. Bold is the preferred model. Θ1=mean one, Θ2=mean 2, σ^2=step variance, μ= step mean, ω=trait variance. LogL=log likelihood, AIC= Akaike information criterion , AW=waited AIC values.

Time bin	Number of specimens	Forms typical of *T. crassaformis*	Percentage abundance
**4.5185**	118	80	67.797
**4.6240**	118	69	58.475
**4.7250**	120	82	68.333
**4.8190**	118	79	66.949
**4.9210**	119	76	63.866
**5.0250**	66	32	48.485
**5.1260**	30	8	26.667
**5.2160**	119	6	5.042
**5.4250**	118	0	0
**5.5250**	97	13	13.402
**5.6240**	120	11	9.167
**5.7290**	76	2	2.632
**5.8130**	120	0	0
**5.8860**	120	1	0.833

Table S4: Changes to abundance of forms typical of *Truncorotalia crassaformis* in morphospace, related to Figure 1 and Figure 2. The forms are very rare before 5.12 Ma (0-13.4%) and increase drastically in samples younger than 5.12 Ma (26.6-68.3%).

Transparent Methods

Samples were derived from washed core microfossil residues from Deep Sea Drilling Project (DSDP) Site 593—consisting of Hole 593 and Hole 593A—on the Lord Howe Rise (40°30'S, 167°40'E; Nelson et al., 1986; Cooke et al., 2008). Both holes were used due to limited material available from Hole 593 alone. Both cores were drilled continuously at the same site from the sea floor at the same time, so represent the same stratigraphic record (Kennett and Von Der Borch, 1986). The abundance of *Truncorotalia* specimens across the Miocene/Pliocene boundary made Holes 593 and 593A ideal for the study. DSDP 593 samples were assigned age values using a time calibration model proposed by Crundwell (see Cooke et al., 2008), which used planktonic foraminiferal bioevents to construct the model and was subsequently calibrated with palaeomagetic records from DSDP Site 1123 and has been applied to DSDP 593 studies in provide age constraint (Crundwell, 2004; Crundwell and Nelson, 2007; Cooke et al., 2008). One residue every *ca*. 100 kyr between 4.5–5.9 Ma was selected using this time calibration and studied. Samples between 5.2–5.9 Ma were Hole 593 residues and samples between 4.5–5.1 Ma were Hole 593A residues. Correlation across the holes was achieved by identifying similarly aged samples about 5.2–5.1 Ma in both holes. Residues were sieved through 300 and 212 µm sieves (to minimize ontogenetic changes in morphology). Where possible, at least 100 standard specimens (no kummerforms, malformed or heavily encrusted specimens) of *Truncorotalia* with a pseudospinose surface ultrastructure and low slit-like apertures were sampled (see taxonomic notes in Scott et al., 1990; 2015; Crundwell and Nelson, 2007). Malformed specimens were not studied as such specimens may have impacted the proposed multivariate analyses and masked the evolutionary signal in question. Specimens were sampled at the genus level to assess change in the genus across the Miocene/Pliocene boundary. The changes in morphology were then linked to the two end member species: *T. juanai* and *T. crassaformis*. However, no species-levels assignments were

made during specimen selection; samples are meant to reflect the bulk of *Truncorotalia* at any given interval.

No standard *Truncorotalia* specimens were identified at 5.3 Ma. A total of 1459 specimens were used in this study. Individuals were mounted in axial view (the most informative view showing morphological change), orientated and imaged using the Automated Measurement System for Shell Morphology (Knappertsbusch et al., 2009; Mary and Knappertsbusch, 2013; 2015; Knappertsbusch, 2016). Outlines of specimen images, as *xy* coordinates, were gathered using the outlining method developed by Knappertsbusch et al. (2009). Outline files were converted into a semilandmark array in R (R Development Core Team, 2017) and outline points were resampled so that all specimens were represented by 200 semilandmarks whose final positions were determined by minimizing their bending energies in Generalized Procrustes Analysis (GPA; Supplementary File 1, Supplementary Data 1). Semilandmark analyses are a useful approach to study foraminiferal evolution, and outlines are the best representation of their morphology (Hull and Norris, 2009; Scott et al., 2015; Hsiang et al., 2016; Shi and Macleod, 2016). However, previous analyses have applied traditional morphometric approaches (e.g., linear measurements, counts of test chambers) and Geometric Morphometric techniques (e.g., Fourier and Eigenshape analyses) (Arnold, 1983; Belyea and Thunell, 1984; Healy-Williams, 1983; 1984; Wei, 1987; Malmgren et al., 1996). Procrustes Superposition and Principal Component Analysis (PCA) were run in the R package geomorph (Adams and Otárola-Castillo, 2013) and visualized through a scatter plot and density distributions (Supplementary Data 2, 3). Temporal variation along Principal Components (PCs) 1 and 2 data were analysed using likelihood-based time-series analyses implemented in the paleoTS package (the 'fit9models' function) to test between various models of evolution (Supplementary Tables 1–3) (Hunt, 2015). Only PCs 1 and 2 (69.9% of the total variance) were interpreted. Variance was not pooled for this analysis. Morphological

variation (i.e., disparity) across our interval was calculated as the Procrustes variance of the GPA-aligned data (Supplementary Data 4). As a result, it is a measure of total disparity and is not limited to specific axes of variation. Disparity was calculated through geomorph. Bimodality was assessed through Hartigan's Dip test for unimodality and analysed for all time bins for PCs 1 and 2 using the diptest package in R (Supplementary Data 5) (Maechler, 2016).

Supplemental References

Adams, D.C., and Otárola-Castillo, E. (2013). geomorph: an r package for the collection and analysis of geometric morphometric shape data. Methods in Ecology and Evolution, 4, 393–399.

Cooke, P.J., Nelson, C.S., and Crundwell, M.P. (2008). Miocene isotope zones, paleotemperatures, and carbon maxima events at intermediate water-depth, Site 593, Southwest Pacific. New Zealand Journal of Geology and Geophysics, 51, 1–22.

Crundwell, M.P. 2004. *New Zealand late Miocene biostratigraphy and biochronology – Studies of planktic foraminifers and bolboforms at oceanic Sites 593 and 1123,, and selected onland sections.* PhD Thesis, University of Waikato, Hamilton, New Zealand.

Crundwell, M.P., and Nelson, C.S. (2007). A magnetostratigraphically-constrained chronology for late Miocene bolboformids and planktic foraminifers in the temperate Southwest Pacific. Stratigraphy, 4, 1–34.

Healy-Williams, N. (1983). Fourier shape analysis of *Globorotalia truncatulinoides* from late Quaternary sediments in the southern Indian Ocean. Marine Micropaleontology, 8, 1–15.

Healy-Williams, N. (1984). Quantitative image analysys: Application to planktonic foraminiferal paleoecology and evolution. Geobios, 17, 425–432.

Hull, P.M., and Norris, R.D. (2009). Evidence for abrupt speciation in a classic case of gradual evolution. Proceedings of the National Academy of Sciences, 106, 21224–21229.

Hunt, G. (2015). paleoTS: Analyze Paleontological Time-Series. R package version 0.5-1.

Knappertsbusch, M. (2016). Evolutionary prospection in the Neogene planktic foraminifer *Globorotalia menardii* and related forms from ODP Hole 925B (Ceara Rise, western tropical Atlantic): evidence for gradual evolution superimposed by long distance dispersal? Swiss Journal of Palaeontology, 135, 205–248.

Knappertsbusch, M., Binggeli, D., Herzig, A., Schmutz, L., Stapfer, S., Schneider, C., Eisenecker, J., and Widmer, L. (2009). AMOR-A new system for automated imaging of microfossils for morphometric analyses. Palaeontologia Electronica, 12.

Maechler, M. (2016). diptest: Hartigan's Dip Test Statistic for Unimodality - Corrected. R package version 0.75-7. https://CRAN.R-project.org/package=diptest.

Mary, Y., and Knappertsbusch, M.W. (2015). Worldwide morphological variability in Mid-Pliocene menardellid globorotalids. Marine Micropaleontology, 121, 1–15.

Mary, Y., and Knappertsbusch, M.W. (2013). Morphological variability of menardiform globorotalids in the Atlantic Ocean during Mid-Pliocene. Marine Micropaleontology, 101, 180–193.

Nelson, C., Hendy, C., and Dudley, W. (1986). Quaternary isotope stratigraphy of Hole 593, Challenger Plateau, south Tasman Sea: preliminary observations based on foraminifers and calcareous nannofossils. Initial reports of the Deep Sea Drilling Project, 90, 1413–1424.

R Development Core Team 2017. R: a language and environment for statistical computing. 3.3.3 ed. Vienna, Austria: R Foundation for Statistical Computing.

Shi, Y., and MacLeod, N. (2016). Identification of life-history stages in fusulinid foraminifera. Marine Micropaleontology, 122, 87–98.
